# Rev-erb Agonist Inhibits Chikungunya and O’nyong’nyong Virus Replication

**DOI:** 10.1093/ofid/ofy315

**Published:** 2018-11-20

**Authors:** Jesse Hwang, Alfred Jiang, Erol Fikrig

**Affiliations:** 1 Section of Infectious Diseases, Department of Internal Medicine, Yale University School of Medicine, New Haven, Connecticut; 2 Howard Hughes Medical Institute, Chevy Chase, Maryland

**Keywords:** Chikungunya, O’nyong’nyong, Rev-erb, antiviral

## Abstract

Chikungunya virus (CHIKV), an alphavirus spread by *Aedes* spp. mosquitos, causes severe inflammation and joint pain, progressing to a chronic arthralgic state in a subset of patients. Due to recent global epidemics of CHIKV and the potential for related viruses to cause outbreaks, multiple approaches to combat these pathogens are of interest. We report that SR9009, a synthetic agonist of nuclear receptors Rev-erb α/β, inhibits replication of multiple alphaviruses (CHIKV and O’nyong’nyong virus) mainly by suppressing structural protein synthesis, although viral RNA accumulation is relatively unimpeded. Furthermore, SR9009 reduces the inflammatory response in cultured murine macrophages exposed to alphavirus-infected cells.

Chikungunya virus (CHIKV) is a mosquito-borne arbovirus that belongs to the alphavirus genus [1]. After mosquito bite, CHIKV replicates locally in the skin, causes viremia, and disseminates to the joints and muscles [2]. Approximately 85% of infected individuals develop symptoms, including rash, fever, and severe joint pain, which are usually self-limiting. Chronic arthralgia, however, can persist for months, and sometimes years, for a subset of patients [2]. There are no approved vaccines or therapeutics against CHIKV.

Although CHIKV normally causes sporadic outbreaks, large epidemics have been documented. From 2004 to 2011, an epidemic that began in Kenya spread to India and Southeast Asia, affecting 1.4–6.5 million people [1]. In 2013, the virus initiated a large epidemic in the Western hemisphere, spreading extensively in the Caribbean and the Americas, with more than 1 million suspected cases [3].

Additional arthritogenic alphaviruses of clinical significance include Sindbis virus, Ross River virus, Mayaro virus, and O’nyong’nyong virus (ONNV) [1]. So far, these alphaviruses have not caused large outbreaks on the scale of CHIKV epidemics. An exception is ONNV, which is transmitted by *Anopheles gambiae* mosquitos and infected more than 2 million people in East Africa from 1959 to 1961 before smoldering out [1, 4].

Viral replication is a spatially and temporally regulated sequence of metabolic reactions within the host cell that results in accumulation of biomass in the form of viral particles that reinitiate the same cycle in the neighboring cell. Thus, manipulating the host cell to render the intracellular environment hostile to the replication cycle is 1 method of inhibiting viruses. Rev-erbα/β belong to the nuclear receptor family of genes and are ubiquitously expressed transcriptional repressors that, upon binding to endogenous heme in the ligand binding domain, recruit corepressors to downregulate target genes involved in circadian rhythm, metabolism, and inflammation [5]. In the liver, Rev-erbα/β prevent metabolic dysfunction and lipid accumulation, whereas in the muscle, Rev-erbα is required to maintain optimal mitochondrial function and exercise endurance [5–7]. The presence of a ligand binding domain allows these factors to be pharmacologically exploited, and synthetic agonists have been developed that target Rev-erbα/β to improve metabolic dysfunction of diet-induced obesity in mice and inhibit inflammatory response in immune cells [6, 7].

As Rev-erbα/β are amenable to pharmacological manipulation both in vitro and in vivo, we tested the effects of Rev-erbα/β ligands on CHIKV and ONNV replication. We report that cultured cells exposed to the agonist SR9009 inhibit alphavirus replication at a late step, namely at the level of viral structural protein accumulation. In addition, SR9009 attenuates alphavirus-triggered inflammatory gene expression in a murine macrophage cell line. To our knowledge, this is the first proof of principle that a synthetic agonist of a host nuclear receptor can inhibit arboviruses.

## METHODS

### Cells, Compounds, and Antibodies

Details are listed in the Supplementary Data.

### Viruses

CHIKV strain 181/25 was obtained from infectious clone plasmid pSinRep5-181/25ic (Addgene: 60078) by generating viral RNA as described previously [8] and transfecting BHK-21 cells using TransIT-mRNA transfection reagent (Mirus Bio).

ONNV strain SG650 and Zika virus strain FSS13025 were obtained from the University of Texas Medical Branch World Reference Center for Emerging Viruses and Arboviruses. Stocks were generated by inoculating Vero cells (ONNV) or C6/36 cells (ZIKV). All virus stocks were stored at –80°C and titered on Vero or C6/36 cells.

For infection, cells were inoculated with virus at indicated multiplicity of infection (MOI) for 1–1.5 hours in a 37°C incubator with occasional rocking. Inoculum was removed, and cells were washed with DMEM and replaced with fresh Dulbecco's Modified Eagle medium (DMEM) +10% fetal bovine serum (FBS) in the presence of compounds or dimethylsulfoxide (DMSO) immediately afterwards. The details of the time-of-addition experiment are supplied in the Supplementary Figure 1 legend.

Virus titer was calculated by 10-fold serial dilutions of infectious media and plaque assay using Vero cells and 1% carboxymethylcellulose overlay.

### Quantative Reverse Transcription Polymerase Chain Reaction Analysis, Immunoblot, Indirect Immunofluorescence Assay, and Statistical Analysis

Details are described in the Supplementary Data.

## RESULTS

**Figure 1.**
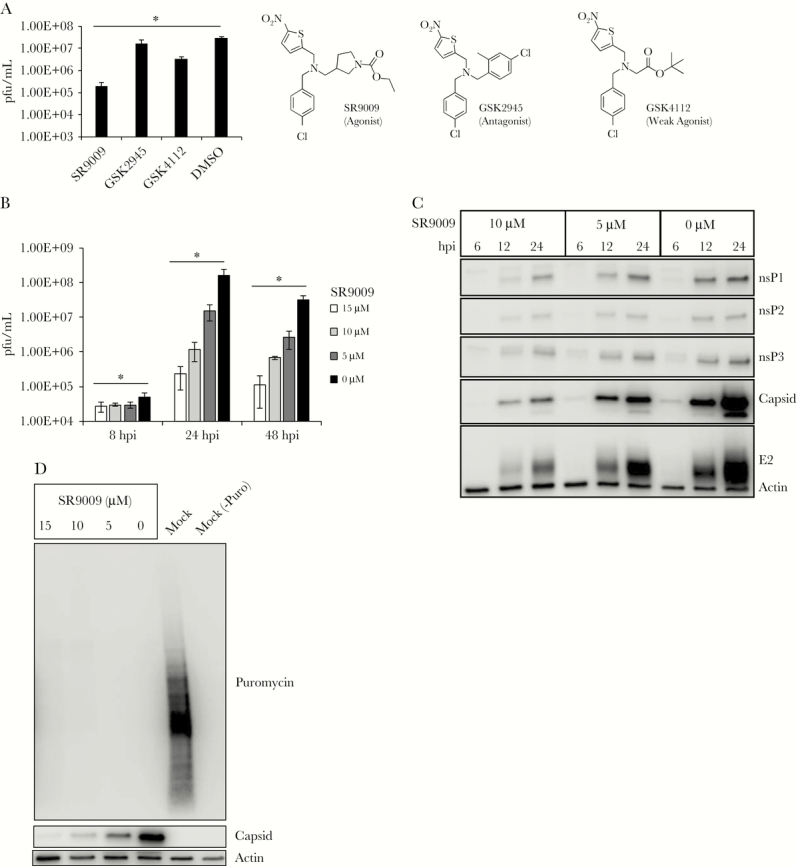
Effect of Rev-erb ligands on Chikungunya virus (CHIKV) replication. A, Huh7 cells were infected with CHIKV (MOI 1) and treated with indicated compounds (10 µM) or DMSO solvent control at 1 hpi. Supernatants were titered at 24 hpi. B, Huh7 cells were infected with CHIKV (MOI 5) and treated with different concentrations of SR9009 at 1 hpi. Supernatants were titered at 8, 24, and 48 hpi. C, Huh7 cells were infected as in (B), and protein lysates were subjected to immunoblot analysis at 6, 12, and 24 hpi. D, Huh7 cells were infected with CHIKV (MOI 5) and treated with SR9009 at 1 hpi. At 23 hpi, puromycin was added to the culture supernatant at a final concentration of 10 µg/mL, and whole-cell lysate was harvested 1 hour later (24 hpi). Protein samples were subjected to immunoblot analysis. Values are the means±SD. Significant values are defined by ^*^P < .05. Abbreviations: DMSO, dimethylsulfoxide; hpi, hour post infection; MOI, multiplicity of infection.

To determine if Rev-erb ligands, reported in the literature to have beneficial in vivo effects in mice [5], can modulate alphavirus replication, we tested 3 structurally similar synthetic compounds. SR9009 is an agonist [6], whereas GSK4112 is a first-generation compound with weaker potency [9]. GSK2945 was reported to have antagonist function [10]. Huh7 cells, which are highly permissive for CHIKV replication, were infected with CHIKV (MOI 1) and treated with the 3 compounds (10 μM) or DMSO. Titration of supernatant at 24 hours postinfection (hpi) showed that GSK4112 led to a 9-fold decrease in viral titer, whereas the effect of GSK2945 was less than 2-fold (Figure 1A). However, the more potent agonist SR9009 led to a >100-fold decrease in infectious progeny (Figure 1A) and exhibited dose-dependent inhibition of viral replication at MOI 5 (Figure 1B).

To identify which step of the infectious cycle is hindered upon application of the agonist, we first analyzed an early event of the viral life cycle, the formation of replication complexes where viral RNA is synthesized. Cells were infected with CHIKV and treated with SR9009 as before. At 12 hpi, cells were processed for indirect immunofluorescence using a monoclonal antibody against double-stranded RNA. We did not observe any striking difference in the localization or level of dsRNA (Supplemental Figure 1A). Quantitative assessment of viral RNA synthesis was performed with quantitative reverse transcription polymerase chain reaction (qRT-PCR) at 12 and 24 hpi. There was no significant difference in the levels of CHIKVE1, and the only statistically significant difference in the level of CHIKVnsP2 was at 12 hpi (Supplementary Figure 1B). Cell viability was not affected as assessed by MTT assay (Supplementary Figure 1C). Thus, viral RNA synthesis and toxicity do not account for the antiviral activities of the Rev-erb ligand.

The alphavirus genome encodes for 2 polyproteins: an early nonstructural polyprotein that becomes sequentially cleaved to carry out viral RNA synthesis and a late structural polyprotein that produces the capsid and envelope proteins [2]. Time-course immunoblot analysis of infected cell lysates showed that the levels of the nonstructural proteins nsP1, nsP2, and nsP3 were slightly lower in cells treated with SR9009 (Figure 1C; Supplementary Figure 2). In contrast to the moderate effects on nonstructural proteins, SR9009 caused a more pronounced reduction of both viral capsid and envelope proteins (Figure 1C). By performing a time-of-addition study, we found that even treating cells beginning at 6 hpi caused dramatic reduction in viral titer (Supplementary Figure 3), consistent with SR9009 inhibiting a relatively late event in the replication cycle. The compound also showed a reduction in viral titer and structural protein levels in infected human foreskin fibroblasts (Supplementary Figure 4).

During the course of alphavirus infection, the translational machinery is altered in such a way that host protein synthesis is halted whereas viral protein synthesis is permitted [11]. To determine if host translational shutoff still occurs in agonist-treated cells, infection was allowed to proceed as described before in the presence of SR9009 or DMSO, and at 23 hpi, puromycin was added to the culture media for 1 hour before harvesting whole-cell lysates. Mock-infected cells incorporated puromycin efficiently, whereas puromycin detection in infected cells was drastically reduced (Figure 1D), consistent with previous reports [11]. Even though SR9009 reduced synthesis of viral capsid protein, there was no proportional increase in puromycin detection. These results show that the early events of the viral life cycle (RNA synthesis and translation shutoff) are not conspicuously perturbed by the compound.

To determine if interferon signaling plays a role, we co-treated cells with SR9009 and ruxolitinib, a Jak inhibitor that efficiently suppresses the interferon signaling pathway, and found no difference in viral titer even at conditions that prevented ISG15 induction (Supplementary Figure 5). We also rule out failure to cleave the polyprotein as a potential mechanism, as there was no accumulation of higher–molecular weight E2E1 protein, and degradation is most likely not the cause of the phenotype, as MG132 (proteasome inhibitor), NMS-873 (ERAD inhibitor), and bafilomycin A (autophagy inhibitor) failed to rescue the levels (Supplementary Figure 6). Moreover, SR9009 does not markedly affect the synthesis of VSV proteins (Supplementary Figure 7). Thus, SR9009 preferentially blocks CHIKV translation.

**Figure 2.**
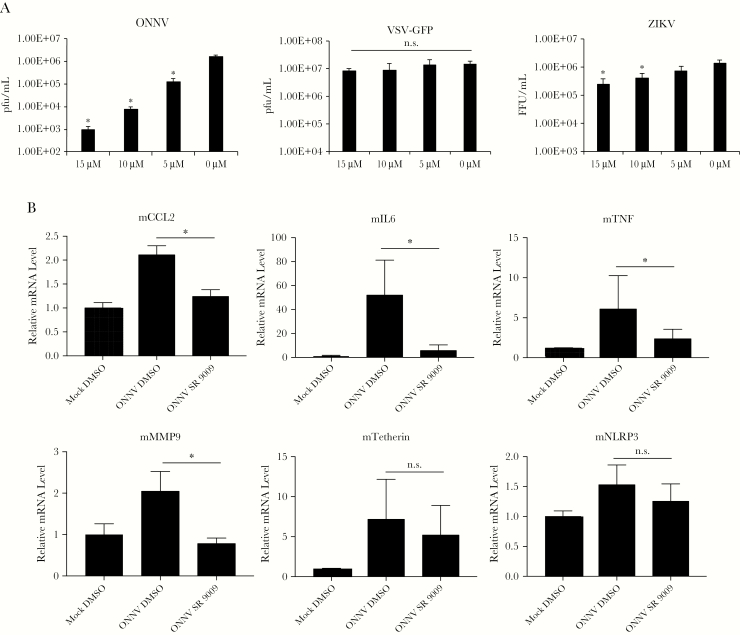
SR9009 preferentially inhibits alphavirus replication and suppresses inflammatory gene expression in murine macrophage cell lines. A, Huh7 cells were infected with O’nyong’nyong virus (ONNV; MOI 5), GFP-expressing vesicular stomatitis virus (VSV-GFP; MOI 0.5), or Zika virus (ZIKV; MOI 3) and treated with increasing doses of SR9009. Supernatants were titered at 24 hpi. B, Huh7 cells were infected with ONNV (MOI 10) or left uninfected. At 6 hpi, cells were trypsinized and seeded on top of Raw264.7 cells pretreated for 1 hour with SR9009 (final = 10 µM) or DMSO. Twenty-four hours later, total RNA was harvested from the co-cultured cells and analyzed by quantitative reverse transcription polymerase chain reaction with murine-specific primers. Values are the means± SD. Significant values are defined by ^*^P < .05.

ONNV is the arthritogenic alphavirus most closely related to CHIKV and has the potential to spread from Africa [4]. Similar to CHIKV, SR9009 reduced ONNV replication > 2 log_10_ fold (Figure 2A). In contrast, SR9009 failed to appreciably inhibit VSV replication and only moderately affected Zika virus replication, with a 6-fold reduction at the highest concentration tested (Figure 2A).

SR9009 has also been shown to downregulate inflammatory gene expression when macrophages are challenged with various tissue damage stimuli [12]. As macrophages play a role in alphavirus-induced arthralgia and infection causes cytopathic effect [1, 13], we sought to determine whether inflammation in macrophages can be subdued by SR9009. Huh7 cells were infected with ONNV (or left uninfected) and seeded on top of murine Raw264.7 macrophage cells that were pretreated for 1 hour with SR9009 or DMSO. The in vitro co-culture simulates contact of macrophages with infected cells. Twenty-four hours after co-culturing, intracellular RNA was harvested for qRT-PCR analysis using primers that only detect murine mRNA. When Raw264.7 cells were co-cultured with ONNV-infected Huh7 cells, there was elevation in mRNA levels of CCL2, IL6, TNFα, and MMP9 that were reduced by SR9009 (Figure 2B). The interferon-stimulated gene Tetherin showed a statistically insignificant downward trend with SR9009 treatment (Figure 2B). These results show that in addition to inhibiting alphavirus replication, Rev-erb agonist may also downregulate host inflammation.

## DISCUSSION

Chikungunya virus circulates in a sylvatic cycle between nonhuman primates and mosquitos in Africa, but spillover infection of humans can lead to an urban cycle and outbreak under the right ecological conditions [1]. Due to the explosive and unpredictable spread of this highly debilitating virus, surveillance is necessary, as well as the identification of pathways that can be manipulated in a targeted manner to block virus multiplication.

Rev-erb α/β are ubiquitously expressed ligand-activated transcriptional repressors with diverse physiological functions, including circadian rhythm, inflammatory response, central carbon metabolism, and lipid homeostasis, and synthetic agonists have been developed that ameliorate metabolic dysfunction in mouse models [5]. We report that the same compound, SR9009, also inhibits alphavirus (CHIKV and ONNV) replication in cultured cells (Figures 1 and 2). Although nonstructural protein levels were slightly decreased, viral RNA accumulation was not significantly altered (Supplementary Figure 1). The most pronounced defect was observed at the level of structural protein synthesis. Host translational shutoff still occurred in the presence of SR9009 (Figure 1D), and there was no accumulation of unprocessed polyprotein or evidence of enhanced degradation (Supplementary Figure 6), suggesting that even though viral translation is poised to be favored after host translation is blocked, subgenomic RNA translation is still inhibited. Although the mechanism is not clear, SR9009 might modulate the composition of the endoplasmic reticulum to hinder proper insertion of viral polyprotein. Alternatively, the ribosomal complex or subunit that is required during alphavirus replication, but not VSV or flavivirus replication, may be altered such that ribosomal loading of RNA or translation initiation is prevented. Further studies are required to pinpoint the mechanism that targets alphaviruses.

Rev-erb α/β also repress inflammatory response in macrophages [5], and this may be physiologically pertinent to alphaviruses, as macrophages are involved in joint damage induced by infection and host response [1, 2, 13]. In an in vitro co-culture system, we show that Rev-erb agonist can suppress macrophage transcript levels of select inflammatory genes (IL6, TNFα, CCL2, and MMP9) (Figure 2B), which are involved in rheumatoid arthritis [14] and implicated in alphavirus-induced joint pathology [15]. Additional studies may be warranted to determine if Rev-erb agonists can suppress arthritogenesis after CHIKV infection in vivo. Plasma levels of SR9009 have been reported to reach up to 20 μM in murine studies [6], near the range we tested in our in vitro studies (10–20 μM).

Repurposing of compounds originally developed for in vivo metabolism studies, such as SR9009, is a promising avenue for identifying potential antiviral therapeutics, but additional work needs to done to establish the mechanism, specificity, and safety. It is conceivable to improve the therapeutic index by utilizing both virus-targeting compounds/antibodies and host-targeting compounds for combinatorial effect to decrease viral burden, pathogenesis, and epidemic spread.

## Supplementary Data

Supplementary materials are available at *Open Forum Infectious Diseases* online. Consisting of data provided by the authors to benefit the reader, the posted materials are not copyedited and are the sole responsibility of the authors, so questions or comments should be addressed to the corresponding author.

ofy315_suppl_supplementary_material
