# Supplementary material for: Rev-erb Agonist Inhibits Chikungunya and O’nyong’nyong Virus Replication
Source: Open Forum Infect Dis. 2018 Nov 20;5(12):ofy315. doi: 10.1093/ofid/ofy315 (PMC6293476; doi:10.1093/ofid/ofy315)
Supplement: ofy315_suppl_supplementary_material [file ofy315_suppl_supplementary_material.docx]

**Supplemental Material**

**Cells, compounds, and antibodies**

Huh7, Raw264.7, and human foreskin fibroblast (HFF) cells expressing hTERT were maintained in DMEM supplemented with 10% FBS and 1% penicillin/streptomycin (Invitrogen). SR9009, GSK4112, and GSK2945 were purchased from Millipore, Cayman Chemicals, and Toronto Research Chemicals, respectively. Stock solutions were dissolved to 10 mM in DMSO and stored at -20° until use.

CHIKV E2 monoclonal antibody (CHK-48), dsRNA J2 antibody, and puromycin monoclonal antibody (4G11) were obtained from BEI Resources, Scicons, and Millipore, respectively. CHIKV Capsid and non-structural protein rabbit polyclonal antibodies were kind gifts from Andre Merits (University of Tartu). Polyclonal antibody against VSV proteins was from Imanis Life Sciences.

**qRT-PCR analysis**

RNA was harvested using the RNeasy kit (Qiagen), and total RNA was reverse transcribed using iScript cDNA synthesis kit (Biorad). Gene expression was assayed using IQ™ SYBR Green Supermix and normalized to 18s rRNA or mHPRT.

**qRT-PCR Primer pairs**

CHIKVE1: 5’-AAACCCGGTAAGAGCGATG-3’, 5’- AGGCTGGTACCTCACACGAC-3’

CHIKVnsP2: 5’-GGCAGTGGTCCCAGATAATTCAAG-3’, 5’-CTGTCTAGATCCACCCCATACATGC-3’

18s rRNA: 5’-GTAACCCGTTGAACCCCATT-3’, 5’-CCATCCAATCGGTAGTAGCG-3’ mIL6: 5’-CTCTGGGAAATCGTGGAAAT-3’, 5’-CCAGTTTGGTAGCATCCATC-3’

mCCL2: 5’-GAAGGAATGGGTCCAGACAT-3’, 5’-ACGGGTCAACTTCACATTCA-3’ mTNF: 5’-TCTTCTCATTCCTGCTTGTGG-3’, 5’-ATGAGAGGGAGGCCATTTG-3’ mTetherin: 5’-AC CAGGAGCTGGAGAATCTG-3’, 5’-ACACTTTGAGCACCAGTAGGC-3’ mHPRT: 5’-TCATTATGCCGAGGATTTGG-3’, 5’-AGAGGGCCACAATGTGATG-3’

mNLRP3: 5’-TCAGGAGTCCAAGTTTTGTGTG-3’, 5’-GGTTTTGAGCACAGAGGTCAG-3’

mMMP9: 5’-TTCGCGTGGATAAGGAGTTC-3’, 5’-TCACACGCCAGAAGAATTTG-3’

**Western blot**

Whole-cell extract was harvested in 1x sample buffer (Biorad) supplemented with 2-mercaptoethanol (2.5%). Protein was analyzed by SDS-PAGE, transfer to nitrocellulose membrane, and incubation with appropriate primary and HRP-conjugated secondary antibodies. ECL reagents (GE Healthcare) were used for detection with Li-Cor Odyssey or Hyblot film.

**Indirect immunofluorescence assay**

Cells were fixed with 2% paraformaldehyde in PBS for 20 minutes at room temperature, permeabilized with PBS + 0.2% Triton X-100 for 10 minutes, and incubated with blocking buffer (PBS + 2% BSA + 0.2% Tween-20) for 30 minutes. Primary J2 monoclonal antibody and secondary goat anti-mouse-CF555 (Biotium) were diluted in blocking buffer and applied to samples for 1 hour each with washing in between steps with PBS + 0.2% Tween-20. Fluorescence microscopy was performed with EVOS imaging system (Thermo Fisher).

**Statistical analysis**

Data analysis was performed using GraphPad. One way ANOVA with Dunnett's multiple comparisons test was used to compare the viral RNA and titer means, and two-way ANOVA with Tukey's multiple comparisons test was used to compare mean gene expression. A value of P < 0.05 was taken to indicate statistical significance.

**Supplemental Figure 1**

Supplemental Figure 1. (A) Huh7 cells were infected with CHIKV and treated with SR9009 (10 µM) or DMSO. Cells were fixed at 12 hpi and processed for indirect immunofluorescence assay. (B) Similar to (A) except total RNA was extracted at 12 hpi and 24 hpi. Viral RNA accumulation was quantified using qRT-PCR analysis and normalized to values obtained from samples collected at 1 hpi (input). (C) Uninfected Huh7 cells were treated with indicated doses of SR9009 in phenol red-free DMEM +10% FBS. MTT assay was carried out 24 hours post treatment using the Vybrant MTT cell proliferation kit (Thermo Fisher). Cells were labeled with MTT for 2 hours and solubilized with DMSO to dissolve the formazan before absorbance was read with a microplate reader. Values are the means ± standard deviation (SD). Significant values are defined by **P* < 0.05.

**Supplemental Figure 2**

Supplemental Figure 2. Huh7 cells were infected with CHIKV (MOI 5) and treated with SR9009 (10 µM) or DMSO at 1 hpi. Protein samples were harvested at 24 hpi for western blot analysis. Semi-quantification of protein levels normalized to actin was performed using Image Studio Software (Licor). Values are the means ± standard deviation (SD). Significant values are defined by **P*< 0.05.

**Supplemental Figure 3**

Supplemental Figure 3. Huh7 cells were pretreated with SR9009 (10 µM) or DMSO for 12 hours and inoculated with CHIKV (MOI 5) for 1 hour. Infected cells were treated with SR9009 (10 µM) or DMSO at the indicated time interval. Supernatants were titered at 24 hpi. Values are the means ± standard deviation (SD). Significant values are defined by **P* < 0.05.

**Supplemental Figure 4**

Supplemental Figure 4. Human foreskin fibroblasts were infected with CHIKV (MOI 1 or 0.01) and treated with SR9009 (20 µM) at 1 hpi. Protein lysates and supernatants were collected at 12 and 24 hpi for western blot analysis and viral titration. Values are the means ± standard deviation (SD). Significant values are defined by **P* < 0.05.

**Supplemental Figure 5**

Supplemental Figure 5. (A) Huh7 cells were incubated with ruxolitinib (2.5 µM) or DMSO for 30 minutes before treating with or without IFNβ (final concentration 10 ng/mL). Ruxolitinib or DMSO remained in the culture media throughout, and protein samples were collected 12 hours later for western blot analysis. (B) Huh7 cells were infected with CHIKV (MOI 3) and treated with SR9009 (10 µM), ruxolitinib (2.5 µM), or DMSO at 1 hpi. Supernatant was titered at 24 hpi.

**Supplemental Figure 6**

Supplemental Figure 6. Huh7 cells were infected with CHIKV (MOI 5) and treated with SR9009 (10 µM) or DMSO at 1 hpi. At 18 hpi, MG132 (final 10 µM), NMS-873 (final 10 µM), bafilomycin A (1 µM), or DMSO were added. Protein lysates were collected 24 hpi.

**Supplemental Figure 7**

Supplemental Figure 7. Huh7 cells were infected with VSV-GFP (MOI 0.5) and treated with SR9009 at 1 hpi. At 24 hpi, protein lysates were harvested for western blot analysis.
